# Alpha-Mangostin in Acute Kidney Injury: Molecular Mechanisms, Regulated Cell Death, and Translational Opportunities

**DOI:** 10.3390/antiox15080915

**Published:** 2026-07-23

**Authors:** Atthaphong Phongphithakchai, Nawanwat C. Pattaranggoon, Kraiyasak Wongna, Ratana Netphakdee, Aman Tedasen, Chutima Jansakun, Wiyada Kwanhian Klangbud, Jongkonnee Thanasai, Fumitaka Kawakami, Moragot Chatatikun

**Affiliations:** 1 Nephrology Unit, Division of Internal Medicine, Faculty of Medicine, Prince of Songkla University, Songkhla 90110, Thailand; atthaphong@psu.ac.th; 2 School of Allied Health Sciences, Walailak University, Nakhon Si Thammarat 80160, Thailand; nawanwat.pa@wu.ac.th (N.C.P.); kaiyasak.wo@mail.wu.ac.th (K.W.); ratana.ne@mail.wu.ac.th (R.N.); aman.te@wu.ac.th (A.T.); chutima.js@wu.ac.th (C.J.); 3 Research Excellence Center for Innovation and Health Products, Walailak University, Tha Sala, Nakhon Si Thammarat 80161, Thailand; 4 Medical Technology Program, Faculty of Science, Nakhon Phanom University, Nakhon Phanom 48000, Thailand; wiyadakwanhian@gmail.com; 5 Faculty of Medicine, Mahasarakham University, Mahasarakham 44000, Thailand; jongkonnee@msu.ac.th; 6 Department of Regulation Biochemistry, Graduate School of Medical Sciences, Kitasato University, Sagamihara 252-0373, Japan; kawakami@kitasato-u.ac.jp; 7 Department of Health Administration, School of Allied Health Sciences, Kitasato University, Sagamihara 252-0373, Japan

**Keywords:** acute kidney injury, alpha-mangostin, oxidative stress, inflammation, apoptosis, mitochondria, translational medicine

## Abstract

Acute kidney injury (AKI) is a major global health challenge associated with substantial morbidity, mortality, and progression to chronic kidney disease. Increasing evidence indicates that oxidative stress, mitochondrial dysfunction, inflammatory signaling, regulated cell death, and maladaptive tissue repair play central roles in AKI pathogenesis, yet effective disease-modifying pharmacological therapies remain unavailable. This narrative review critically evaluated current evidence regarding the pharmacological characteristics, molecular mechanisms, and translational potential of alpha-mangostin (AM), the principal prenylated xanthone isolated from the pericarp of *Garcinia mangostana* L., through a comprehensive synthesis of experimental and mechanistic studies. Available preclinical evidence consistently demonstrates that AM improves renal function and attenuates histopathological injury, particularly in cisplatin-induced nephrotoxicity and glycerol-induced rhabdomyolysis models. These renoprotective effects are primarily associated with suppression of oxidative stress, activation of the Nrf2/HO-1 antioxidant pathway, inhibition of NF-κB-mediated inflammatory signaling, preservation of mitochondrial function, and attenuation of apoptosis. Several emerging pathways may also contribute to AM-mediated renoprotective effects; however, current evidence remains indirect, and their roles require validation in kidney-specific models. Clinical translation remains limited by poor oral bioavailability, insufficient pharmacokinetic data, lack of standardized formulations, and the absence of human clinical trials. Overall, current evidence suggests that AM has preliminary renoprotective potential in experimental AKI models. However, the limited number of available studies, predominance of cisplatin-induced nephrotoxicity models, insufficient pharmacokinetic and safety data, and absence of human clinical studies preclude conclusions regarding its clinical efficacy or translational readiness. Further validation in diverse and clinically relevant AKI models is required before clinical investigation can be considered.

## 1. Introduction

Acute kidney injury (AKI) is characterized by an abrupt decline in kidney function occurring within hours to days and remains one of the most common complications among hospitalized and critically ill patients [1]. Depending on patient populations and diagnostic criteria, AKI affects approximately 10–20% of hospitalized patients and more than 50% of patients admitted to intensive care units [2,3]. Despite advances in supportive management, AKI continues to be associated with high short-term mortality, prolonged hospitalization, increased healthcare costs, and progression to chronic kidney disease (CKD) and end-stage kidney disease [3,4].

The pathogenesis of AKI is highly complex and involves multiple interconnected biological processes rather than a single pathological pathway [3,5]. Renal ischemia, nephrotoxins, sepsis, and rhabdomyolysis initiate excessive production of reactive oxygen species (ROS), mitochondrial dysfunction, endothelial injury, inflammatory activation, tubular epithelial cell death, and maladaptive repair [5,6]. Crosstalk among these mechanisms amplifies tissue injury and contributes to persistent renal dysfunction [3,5]. Consequently, therapeutic strategies targeting only one downstream pathway have generally failed to demonstrate consistent clinical benefit [3].

Oxidative stress is increasingly recognized as a central driver of AKI progression [5,7]. Excessive ROS damages proteins, lipids, and DNA while simultaneously activating inflammatory cascades through nuclear factor-kappa B (NF-κB), mitogen-activated protein kinase (MAPK), and nucleotide-binding oligomerization domain-like receptor family pyrin domain-containing 3 (NLRP3) inflammasome signaling [5,8]. Persistent oxidative injury further induces mitochondrial dysfunction, ATP depletion, apoptosis, ferroptosis, and pyroptosis, ultimately leading to extensive tubular epithelial injury and impaired renal recovery [5,8,9].

Natural bioactive compounds have recently attracted considerable attention because they can simultaneously modulate multiple pathogenic pathways involved in AKI [10]. Among these compounds, alpha-mangostin (AM), the major xanthone isolated from the pericarp of *Garcinia mangostana* L., possesses potent antioxidant, anti-inflammatory, anti-apoptotic, antimicrobial, and anti-fibrotic activities [10,11,12]. Experimental studies have demonstrated that AM attenuates renal injury in various AKI models by reducing oxidative stress, suppressing inflammatory cytokines, preserving mitochondrial function, and improving renal histopathology [11,12,13]. A recent systematic review and meta-analysis further confirmed the nephroprotective effects of AM across experimental AKI models, demonstrating significant improvements in serum creatinine, blood urea nitrogen, oxidative stress biomarkers, inflammatory mediators, and renal histological injury [14]. Nevertheless, current evidence remains almost exclusively derived from animal studies [11,12,13,14]. Moreover, several emerging mechanisms including ferroptosis, pyroptosis, mitochondrial quality control, kidney repair, and immunometabolism have not yet been comprehensively discussed in previous reviews [5,9].

**Figure 1:**
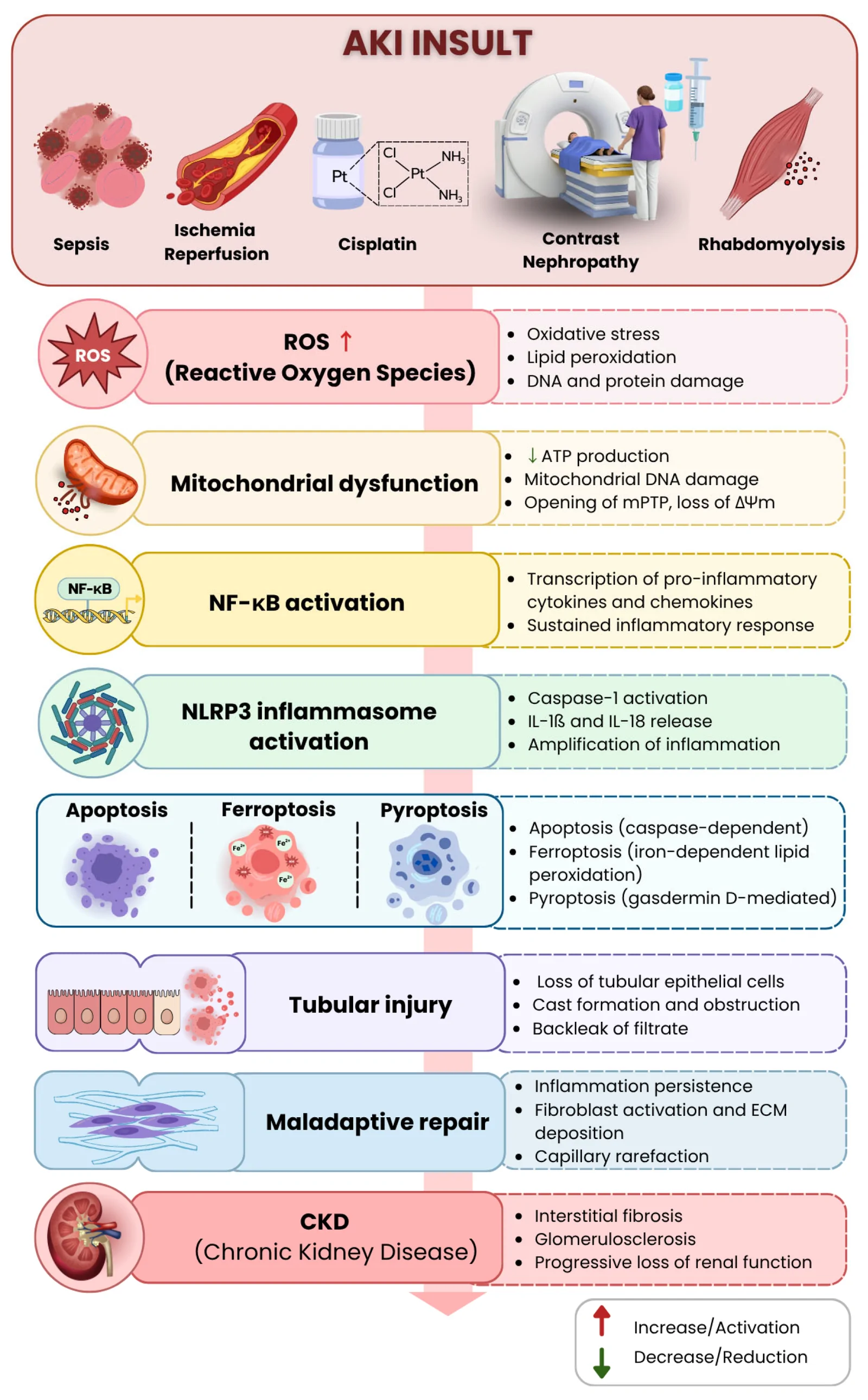
Pathophysiology of acute kidney injury and potential therapeutic targets of AM.

Therefore, this narrative review aims to critically summarize the current evidence regarding the pharmacological properties and potential molecular mechanisms of AM in experimental AKI, while highlighting emerging areas involving regulated cell death, mitochondrial biology, and translational research. Rather than considering AM solely as an antioxidant, we examine its potential to modulate multiple interconnected pathways involved in AKI pathogenesis and renal repair. However, given the limited number of available experimental studies and the absence of human clinical data, these proposed multi-target effects should be regarded as preliminary and hypothesis-generating rather than evidence of clinical efficacy or translational readiness. An overview of the interconnected molecular pathways involved in AKI pathogenesis and the potential sites of action of AM is illustrated in Figure 1.

## 2. Pathophysiology of Acute Kidney Injury

Recent advances in molecular nephrology have substantially changed our understanding of AKI. Instead of considering tubular necrosis as the predominant pathological event, current evidence suggests that tubular epithelial cells undergo highly regulated biological responses involving stress adaptation, mitochondrial remodeling, inflammatory signaling, programmed cell death, and cellular senescence [6,7,8,15]. Consequently, therapeutic agents capable of simultaneously modulating multiple pathogenic pathways are increasingly considered more promising than drugs targeting a single signaling molecule [6,7].

### 2.1. Oxidative Stress: The Initial Trigger of Renal Injury

Oxidative stress is widely recognized as one of the earliest events following renal ischemia or toxic injury [7,8]. Under physiological conditions, ROS generated by mitochondria participate in intracellular signaling and cellular homeostasis. However, excessive ROS production overwhelms endogenous antioxidant defenses, leading to oxidative damage of proteins, membrane lipids, and nucleic acids [7,8,16,17]. During AKI, ROS originate from multiple intracellular sources, including dysfunctional mitochondria, NADPH oxidase (NOX), xanthine oxidase, uncoupled nitric oxide synthase, and activated inflammatory cells [7,16]. Accumulation of superoxide anions and hydrogen peroxide promotes lipid peroxidation, protein carbonylation, DNA fragmentation, and mitochondrial DNA damage, ultimately impairing tubular epithelial cell survival [16,17].

The transcription factor nuclear factor erythroid 2-related factor 2 (Nrf2) constitutes one of the principal endogenous antioxidant defense systems [18]. Under oxidative stress, Nrf2 dissociates from Kelch-like ECH-associated protein 1 (Keap), translocates into the nucleus, and induces transcription of antioxidant enzymes including heme oxygenase-1 (HO-1), superoxide dismutase (SOD), glutathione peroxidase (GPx), catalase, and NAD(P)H quinone oxidoreductase-1 (NQO1) [18,19,20]. Pharmacological activation of the Nrf2/HO-1 pathway has consistently demonstrated renoprotective effects in experimental AKI models [19,20].

### 2.2. Inflammatory Response and Innate Immunity

Following oxidative injury, damaged tubular epithelial cells release damage-associated molecular patterns (DAMPs), which activate Toll-like receptors (TLRs) and pattern-recognition receptors expressed by macrophages, dendritic cells, and renal tubular cells [6,21]. Activation of these receptors stimulates downstream NF-κB signaling, resulting in increased production of tumor necrosis factor-alpha (TNF-α), interleukin (IL)-1β, IL-6, monocyte chemoattractant protein-1 (MCP-1) and various chemokines that recruit neutrophils and monocytes into injured renal tissue [21,22]. Persistent inflammatory activation contributes not only to acute tubular damage but also to maladaptive tissue remodeling and subsequent progression toward chronic kidney disease [22]. Among inflammatory mediators, the NLRP3 inflammasome has emerged as a central regulator of AKI pathogenesis. Activation of NLRP3 induces cleavage of caspase-1 and maturation of IL-1β and IL-18, thereby amplifying inflammatory injury and promoting pyroptotic cell death [23,24,25]. Experimental inhibition of NF-κB or NLRP3 signaling significantly attenuates renal injury in animal models, highlighting these pathways as attractive therapeutic targets [23,25].

### 2.3. Mitochondrial Dysfunction and Bioenergetic Failure

Renal proximal tubular epithelial cells possess exceptionally high mitochondrial density because of their dependence on oxidative phosphorylation for ATP production [9,21]. Consequently, mitochondrial dysfunction rapidly compromises tubular transport, cytoskeletal integrity, and cellular viability during AKI [26,27]. Excessive ROS disrupt mitochondrial membrane potential, impair electron transport chain activity, reduce ATP synthesis, and trigger opening of the mitochondrial permeability transition pore [26,27,28]. Damaged mitochondria subsequently release cytochrome c, mitochondrial DNA, and other danger signals that further amplify inflammatory responses and apoptosis [27,28]. Maintenance of mitochondrial quality through mitophagy has emerged as a critical adaptive mechanism during AKI. The PTEN-induced kinase 1 (PINK1)/Parkin pathway selectively removes damaged mitochondria and preserves cellular homeostasis [27,29]. Failure of mitochondrial quality control exacerbates oxidative stress, inflammation, and tubular cell death, thereby delaying renal recovery [27,28,29].

### 2.4. Regulated Cell Death in AKI

Accumulating evidence indicates that multiple forms of regulated cell death coexist in AKI [9,15,25]. Apoptosis is characterized by mitochondrial cytochrome c release, activation of caspase-9 and caspase-3, DNA fragmentation, and cell shrinkage [9]. In contrast, necroptosis depends on receptor-interacting protein kinase-1 (RIPK1), RIPK3, and mixed lineage kinase domain-like protein (MLKL), resulting in plasma membrane rupture and inflammatory mediator release [9,15]. More recently, ferroptosis has gained considerable attention because it is driven by iron-dependent lipid peroxidation and depletion of glutathione peroxidase 4 (GPX4) [30,31]. Inhibition of ferroptosis markedly reduces experimental AKI severity, suggesting an important therapeutic opportunity [30]. Pyroptosis represents another inflammatory form of programmed cell death mediated by inflammasome activation, caspase-1 cleavage, and gasdermin D pore formation [24,25]. Emerging evidence indicates that pyroptosis contributes significantly to septic AKI and ischemia–reperfusion injury [31]. Because oxidative stress, mitochondrial dysfunction, inflammation, apoptosis, ferroptosis, and pyroptosis are tightly interconnected, therapies capable of simultaneously modulating several of these pathways may provide greater renoprotection than agents acting on a single molecular target. AM has been reported to influence multiple components of this network, making it an attractive candidate for translational investigation in AKI.

## 3. Pharmacological Characteristics of AM

### 3.1. Natural Source and Chemical Structure

AM is the major prenylated xanthone isolated from the pericarp of mangosteen (*Garcinia mangostana* L.), a tropical fruit widely cultivated in Southeast Asia [32,33,34,35]. Among more than 70 naturally occurring xanthones identified in mangosteen, AM represents the predominant bioactive constituent and accounts for a substantial proportion of the total xanthone content within the fruit pericarp [32,33]. Traditionally, mangosteen has been used in folk medicine for the treatment of skin infections, wounds, dysentery, inflammation, and gastrointestinal disorders, suggesting a long history of medicinal application prior to modern pharmacological investigation [33,36]. More recently, AM has attracted increasing attention because of its broad spectrum of biological activities, including antioxidant, anti-inflammatory, antimicrobial, anticancer, antidiabetic, cardioprotective, neuroprotective, and renoprotective effects [33,34,35].

**Figure 2:**
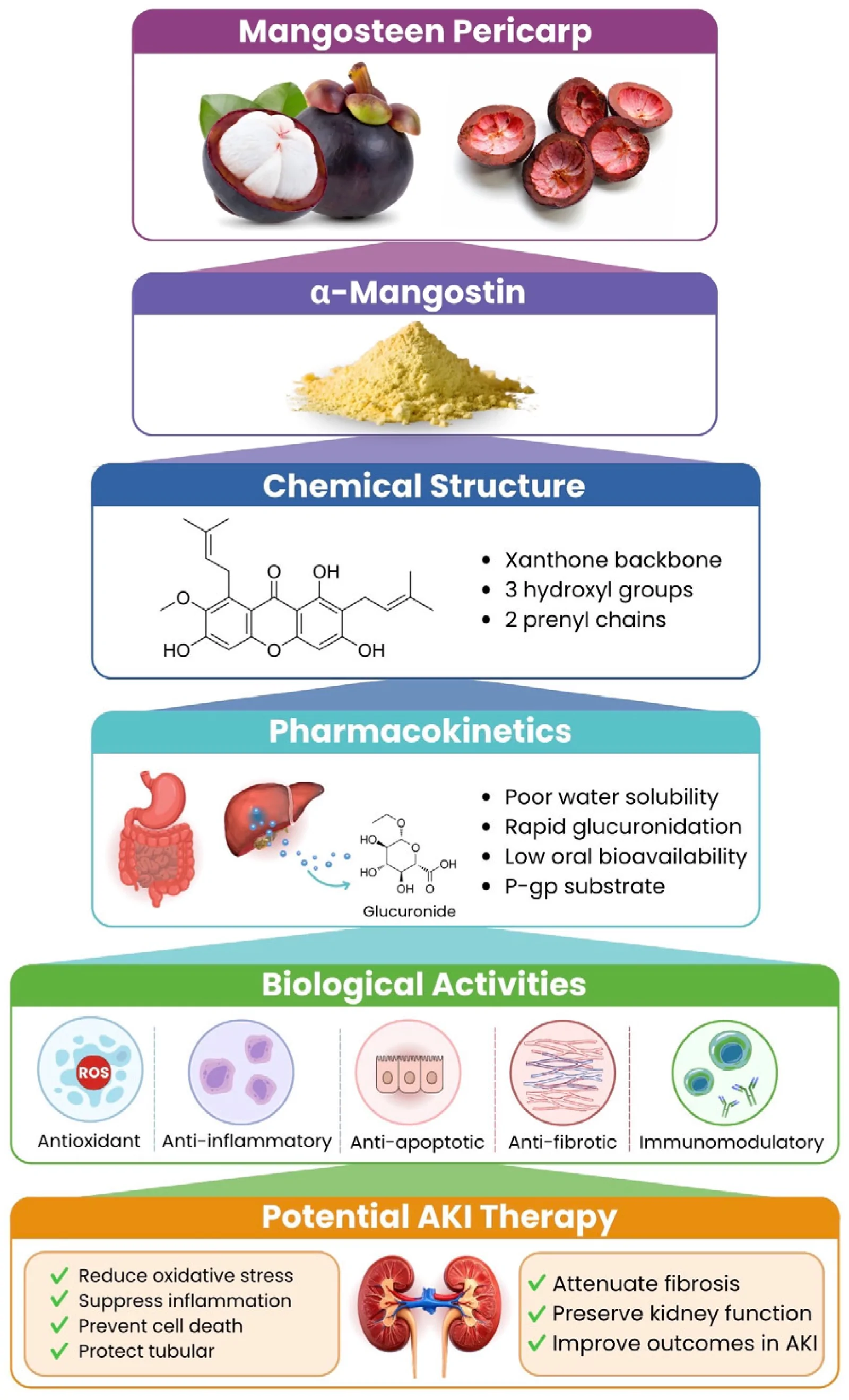
Chemical and pharmacological characteristics of AM.

Structurally, AM is chemically designated as 1,3,6-trihydroxy-7-methoxy-2,8-bis (3-methyl-2-butenyl)-9H-xanthen-9-one. The molecule consists of a tricyclic xanthone backbone containing three hydroxyl groups, one methoxy group, and two prenyl side chains [32,33]. This unique structure confers remarkable free radical scavenging capacity while simultaneously facilitating interactions with multiple intracellular signaling proteins involved in oxidative stress and inflammatory responses [32,34]. The hydroxyl groups contribute to antioxidant activity through hydrogen donation, whereas the prenyl substituents increase lipophilicity, allowing efficient membrane penetration and intracellular accumulation [32,37]. Recent computational studies further suggest that AM exhibits favorable binding affinity toward numerous therapeutic targets, including NF-κB, NLRP3 inflammasome-related proteins, PI3K/Akt, MAPKs, cyclooxygenase-2 (COX-2), inducible nitric oxide synthase (iNOS), and several apoptosis-regulating proteins [34]. These findings support the concept that AM functions as a multi-target therapeutic compound rather than a single-pathway inhibitor. The chemical origin, structural characteristics, pharmacokinetic properties, and principal biological activities of AM are summarized in Figure 2.

### 3.2. Pharmacokinetic Properties

Despite promising pharmacological activities, clinical translation of AM remains limited by several unfavorable pharmacokinetic characteristics [32,34]. The molecule is highly lipophilic and exhibits extremely poor aqueous solubility, resulting in limited oral absorption and variable systemic bioavailability [34,37]. In experimental pharmacokinetic studies, orally administered AM reached peak plasma concentrations within approximately 30–60 min, followed by relatively rapid elimination with extensive phase II metabolism, particularly glucuronidation [37]. Microsomal studies have demonstrated that glucuronide conjugation represents the principal metabolic pathway, whereas CYP 450-mediated phase I metabolism appears to play a relatively minor role [37]. Furthermore, AM has been proposed as a substrate of P-glycoprotein (P-gp), which may further restrict intestinal absorption by promoting active efflux from enterocytes into the intestinal lumen [37]. These pharmacokinetic limitations may explain why the remarkable efficacy observed in experimental AKI models has not yet translated into clinical application. Several formulation strategies have therefore been investigated to improve systemic exposure. Liposomal formulations, polymeric nanoparticles, solid lipid nanoparticles, nanoemulsions, and phospholipid complexes have all demonstrated improved solubility, prolonged circulation time, and enhanced tissue delivery in preclinical studies [34,38]. Such approaches may represent critical steps toward future clinical translation of AM for kidney diseases.

### 3.3. Safety Profile

The safety profile of AM remains incompletely characterized. Although several animal studies have reported acceptable tolerability at selected doses and treatment durations, findings vary across experimental models, formulations, routes of administration, and exposure periods [39]. Importantly, the absence of overt toxicity in short-term animal studies does not establish long-term safety or define a clinically relevant therapeutic window. Available toxicological evidence suggests that adverse effects may be dose- and exposure-dependent, emphasizing the need to distinguish pharmacologically active doses from potentially toxic concentrations [39]. The broad biological activity of AM may also result in off-target effects. In addition to modulating oxidative stress and inflammatory signaling, AM interacts with multiple molecular pathways and may influence drug-metabolizing enzymes and membrane transporters [34,37]. Such effects could alter the pharmacokinetics of concomitant medications and may be particularly relevant in patients with AKI, who commonly receive multiple drugs and have rapidly changing kidney and hepatic function. Furthermore, the high lipophilicity of AM and its potential for tissue accumulation raise additional concerns regarding prolonged or repeated exposure, although organ-specific distribution and toxicity remain insufficiently characterized. Current evidence is also limited by substantial heterogeneity in AM formulations, doses, routes of administration, and treatment durations. Long-term toxicity, reproductive and developmental toxicity, genotoxicity, immunotoxicity, kidney-specific toxicity, and drug–drug interactions remain inadequately studied. Moreover, safety findings from mangosteen extracts cannot necessarily be extrapolated to purified AM because of differences in composition, bioavailability, and exposure. Although limited human studies of mangosteen-derived products have not identified major short-term safety concerns, these findings do not establish the safety of purified AM at therapeutic doses or in patients with AKI [39,40]. Therefore, comprehensive dose–response and repeated-dose toxicity studies, together with pharmacokinetic–toxicodynamic analyses, are required to define the therapeutic window, identify potential target-organ toxicity, and characterize clinically relevant off-target and drug–drug interactions before AM can be evaluated as a therapeutic intervention for AKI.

### 3.4. Why AM Is an Attractive Candidate for AKI

The pathogenesis of AKI involves multiple interacting mechanisms rather than a single pathogenic pathway. Oxidative stress promotes inflammatory activation, which further induces mitochondrial dysfunction, apoptosis, ferroptosis, endothelial injury, and maladaptive tissue repair [3,4,5,6,7,8,9]. Therefore, pharmacological agents capable of simultaneously modulating several interconnected pathways may provide greater therapeutic efficacy than drugs targeting only one molecular mechanism. AM appears particularly attractive because its reported biological activities overlap remarkably well with the principal mechanisms responsible for AKI progression. Experimental studies consistently demonstrate suppression of oxidative stress through activation of the Nrf2/HO-1 pathway, inhibition of NF-κB-mediated inflammatory signaling, preservation of mitochondrial integrity, attenuation of apoptotic signaling, and reduction in inflammatory cytokine production [11,12,18]. More recent evidence also suggests potential regulation of mitochondrial quality control and inflammasome activation, although these mechanisms require further investigation in kidney-specific experimental models [12,34,38].

Collectively, these findings support the hypothesis that AM should be regarded not merely as an antioxidant phytochemical but rather as a multi-target molecular modulator capable of influencing the complex signaling networks underlying AKI. This concept provides the mechanistic rationale for the subsequent sections of this review, which critically examine experimental evidence supporting its renoprotective effects.

## 4. Molecular Mechanisms Underlying the Renoprotective Effects of Am

### 4.1. Modulation of Oxidative Stress Through the Nrf2/HO-1 Signaling Pathway

Oxidative stress is widely regarded as a pivotal mechanism driving the initiation and progression of AKI. Excessive production of reactive oxygen species (ROS) damages membrane lipids, proteins, and nucleic acids while impairing mitochondrial function and ATP generation [3,7,8]. These events subsequently activate inflammatory cascades and multiple forms of regulated cell death, thereby amplifying renal injury [3,7]. Among endogenous antioxidant defense systems, the Nrf2 pathway represents one of the most important cytoprotective mechanisms [18,19,20]. Under physiological conditions, Nrf2 is retained in the cytoplasm by Keap1. During oxidative stress, dissociation of the Nrf2-Keap1 complex permits nuclear translocation of Nrf2, which induces transcription of numerous antioxidant enzymes, including HO-1, NAD(P)H quinone oxidoreductase-1 (NQO1), GPx, SOD, catalase, and glutamate-cysteine ligase [18,19,20].

Experimental studies indicate that AM enhances endogenous antioxidant capacity through activation of the Nrf2/HO-1 signaling pathway [12,13,14]. In cisplatin-induced AKI models, treatment with AM significantly reduced renal malondialdehyde (MDA) concentrations while restoring antioxidant enzyme activity, including SOD, catalase, and glutathione levels [11,12]. These biochemical improvements were accompanied by attenuation of tubular necrosis and preservation of renal function [11]. Beyond direct free-radical scavenging, AM appears to influence upstream regulators of oxidative stress by suppressing ROS generation and preserving mitochondrial integrity [12,34]. Consequently, AM should be regarded as a modulator of oxidative homeostasis rather than merely a conventional antioxidant molecule.

### 4.2. Suppression of NF-κB-Mediated Inflammatory Signaling

Inflammation represents a second major pathogenic component of AKI and is closely interconnected with oxidative stress [6,22]. Reactive oxygen species activate NF-κB, which subsequently promotes transcription of multiple pro-inflammatory cytokines, including TNF-α, IL-1β, IL-6, iNOS, COX-2, and MCP-1 [22,34].

Several experimental investigations consistently demonstrate that AM suppresses activation of the NF-κB pathway [11,12,34]. Reduced nuclear translocation of NF-κB is associated with lower circulating concentrations of TNF-α, IL-1β, and IL-6 together with decreased inflammatory cell infiltration into injured renal tissue [19,34]. In glycerol-induced rhabdomyolysis, AM treatment significantly reduced systemic inflammatory cytokines and improved renal histopathological injury, supporting the concept that inhibition of inflammatory amplification contributes substantially to its renoprotective activity [19]. Because oxidative stress and inflammation form a positive feedback loop during AKI, simultaneous suppression of both pathways may explain why AM consistently demonstrates greater efficacy than would be expected from antioxidant activity alone.

### 4.3. Preservation of Mitochondrial Function

Renal proximal tubular epithelial cells require continuous ATP production to maintain sodium transport and cellular homeostasis [26]. Mitochondrial injury therefore rapidly compromises tubular function during AKI [26,27,28]. Mitochondrial dysfunction results in collapse of membrane potential, impaired oxidative phosphorylation, ATP depletion, release of cytochrome c, and excessive mitochondrial ROS generation [26,27,28]. These alterations initiate apoptosis while simultaneously amplifying inflammatory signaling through release of mitochondrial danger-associated molecular patterns [27]. Reyes-Fermín and colleagues demonstrated that AM preserved mitochondrial membrane potential, reduced mitochondrial oxidative injury, and improved ATP-dependent cellular function in cisplatin nephrotoxicity [12]. These findings suggest that mitochondrial preservation represents one of the principal mechanisms underlying the nephroprotective effects of AM. Recent systems pharmacology analyses further suggest possible interactions between AM and signaling pathways involved in mitochondrial quality control, including PI3K/Akt and AMP-activated protein kinase (AMPK) [34]. However, direct evidence regarding regulation of PINK1/Parkin-mediated mitophagy in AKI remains limited and warrants further investigation.

### 4.4. Attenuation of Apoptotic Signaling

Apoptosis contributes to tubular epithelial cell injury during AKI. Activation of the intrinsic mitochondrial apoptotic pathway involves mitochondrial cytochrome c release, caspase-9 activation, downstream caspase-3 cleavage, and DNA fragmentation [16,26]. In experimental cisplatin-induced renal cell injury, AM treatment has been associated with changes in apoptosis-related markers, including increased Bcl-2 expression, reduced Bax expression, and decreased cleavage of caspase-3 and PARP [12]. These findings are consistent with attenuation of apoptotic signaling but do not establish direct molecular interactions between AM and individual apoptotic proteins. Because apoptosis is closely linked to oxidative stress and mitochondrial dysfunction, the observed changes in apoptotic markers may reflect indirect downstream effects of reduced ROS production, preservation of mitochondrial function, or modulation of upstream signaling pathways, including PI3K/Akt and JNK [12]. Further studies using target-specific approaches are required to determine whether AM directly modulates apoptotic proteins or indirectly alters their expression and activation through upstream cytoprotective mechanisms.

### 4.5. Proposed Emerging Mechanisms: Ferroptosis and Pyroptosis

In contrast to the experimentally supported effects of AM on oxidative stress, inflammatory signaling, mitochondrial preservation, and apoptosis, its effects on several emerging pathways involved in AKI remain incompletely defined. Direct kidney-specific evidence linking AM to ferroptosis, pyroptosis, PANoptosis, immunometabolism, and cellular senescence is currently limited or absent. Therefore, these pathways should be regarded as mechanistically plausible hypotheses and priorities for future investigation rather than established mechanisms of AM-mediated renoprotective effects.

Interest in regulated cell death has expanded rapidly during the past decade, particularly regarding ferroptosis and pyroptosis [11,23,25]. Ferroptosis is characterized by iron-dependent lipid peroxidation resulting from depletion of GPX4 [11]. Increasing evidence indicates that ferroptosis contributes to ischemia–reperfusion injury, cisplatin nephrotoxicity, and septic AKI [11]. Although direct experimental evidence linking AM with ferroptosis inhibition in AKI remains limited, its potent antioxidant activity, preservation of glutathione homeostasis, and attenuation of lipid peroxidation strongly support the hypothesis that modulation of ferroptotic pathways may contribute to renal protection [12,34]. Future studies should investigate GPX4 expression, iron metabolism, and lipid peroxide accumulation following AM treatment.

Pyroptosis is another inflammatory form of regulated cell death mediated through activation of the NLRP3 inflammasome, caspase-1 cleavage, and gasdermin D pore formation [23,25]. Activation of this pathway promotes maturation of IL-1β and IL-18 and further amplifies renal inflammation [23]. Experimental evidence outside nephrology indicates that AM suppresses NLRP3 inflammasome activation in several inflammatory diseases [34]. However, kidney-specific evidence remains scarce, representing an important knowledge gap and a promising direction for future mechanistic studies.

### 4.6. An Integrated Mechanistic Model

**Figure 3:**
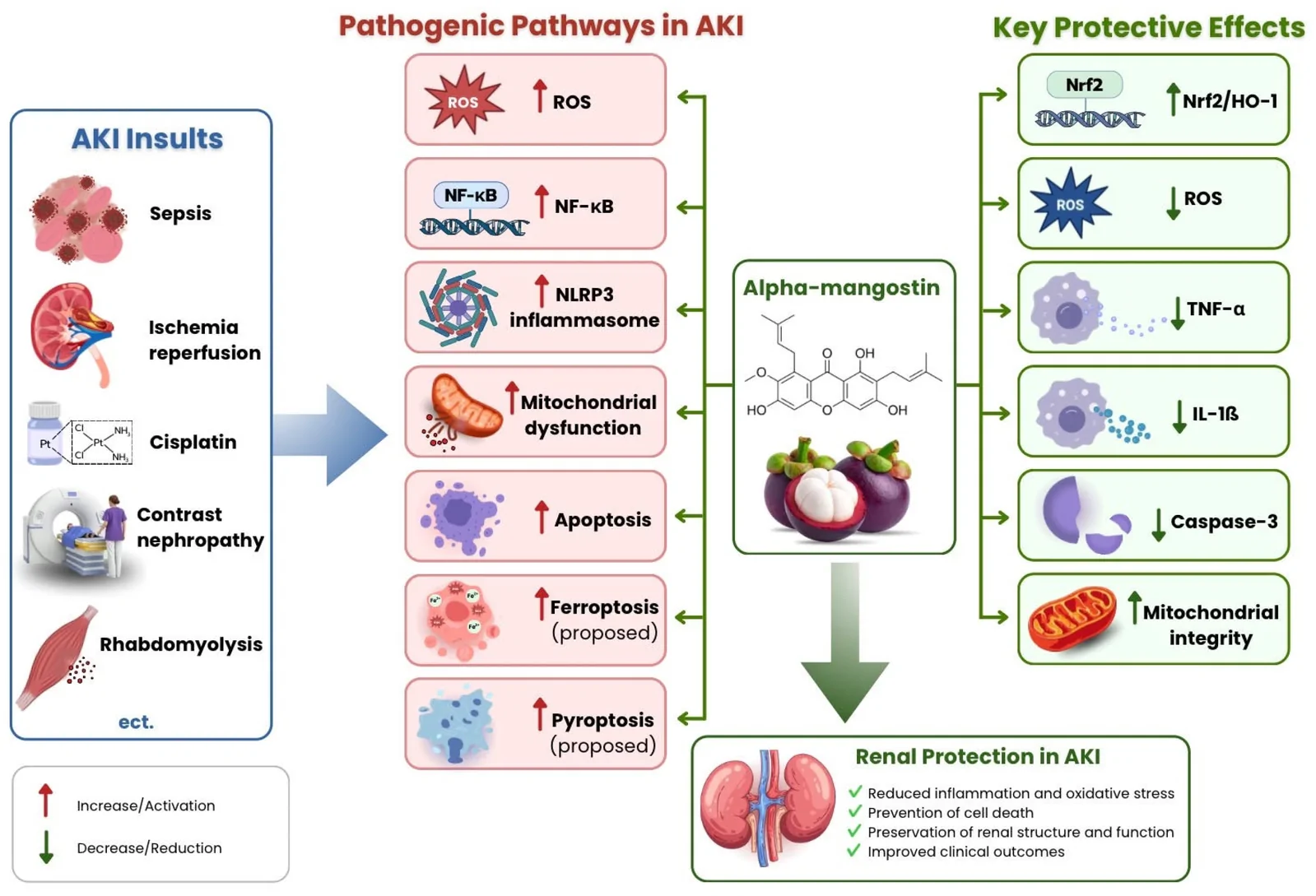
Integrated molecular mechanisms of AM in acute kidney injury.

Collectively, the current evidence supports a multi-target model in which AM interrupts several interconnected pathogenic pathways simultaneously. Initial suppression of oxidative stress reduces mitochondrial injury, thereby limiting NF-κB activation, inflammatory cytokine production, apoptotic signaling, and possibly ferroptotic and pyroptotic cell death. Rather than functioning through a single molecular target, AM appears to restore intracellular homeostasis by modulating multiple biological networks involved in AKI pathogenesis. This integrated mechanistic perspective provides a strong rationale for further translational studies and supports continued investigation of AM as a candidate disease-modifying therapy for AKI. The integrated molecular mechanisms underlying the renoprotective effects of -AM are illustrated in Figure 3.

## 5. Evidence from Experimental Models of Acute Kidney Injury

### 5.1. Overview of Experimental Evidence

To date, evidence supporting the renoprotective effects of AM has been derived almost exclusively from experimental studies using both in vivo and in vitro models of AKI. Collectively, these studies consistently demonstrate improvements in renal function, attenuation of tubular injury, reduction in oxidative stress, and suppression of inflammatory responses following AM administration [11,12,14,41]. Currently available evidence is derived primarily from cisplatin-induced nephrotoxicity, glycerol-induced rhabdomyolysis, and renal tubular cell injury models. Although direct evidence in ischemia–reperfusion and sepsis-associated AKI remains limited, these models share common downstream mechanisms involving oxidative stress, mitochondrial dysfunction, inflammatory activation, and tubular epithelial cell death [3,14,42]. Although these experimental models share several downstream mechanisms, including oxidative stress, mitochondrial dysfunction, inflammatory activation, and tubular epithelial cell death, the limited number and diversity of available studies preclude firm conclusions regarding the consistency or generalizability of AM-mediated renoprotective effects across different AKI etiologies. The observed effects should therefore be considered preliminary and require independent validation in additional clinically relevant models, particularly sepsis-associated and ischemia–reperfusion AKI. The principal characteristics, experimental models, and major findings of published studies are summarized in Table 1.

### 5.2. Cisplatin-Induced Acute Kidney Injury

Cisplatin nephrotoxicity remains the most extensively investigated experimental model evaluating the renoprotective effects of AM. Cisplatin accumulates within proximal tubular epithelial cells through organic cation transporters, inducing mitochondrial dysfunction, excessive reactive oxygen species (ROS) production, DNA damage, inflammatory activation, and apoptosis [11,12,14]. Pérez-Rojas and colleagues first demonstrated that AM significantly attenuated cisplatin-induced nephrotoxicity in rats by reducing oxidative and nitrosative stress while improving renal function and histological injury. Treatment with AM reduced serum creatinine and blood urea nitrogen (BUN), preserved renal glutathione concentrations, decreased lipid peroxidation, and attenuated tubular necrosis, indicating substantial protection against oxidative damage [11]. Subsequently, Reyes-Fermín et al. extended these observations by demonstrating that mitochondrial preservation represents another major mechanism underlying AM-mediated renoprotective effects. In LLC-PK1 proximal tubular cells exposed to cisplatin, AM preserved mitochondrial respiration, maintained oxidative phosphorylation, prevented mitochondrial membrane depolarization, and reduced excessive mitophagy. Furthermore, AM restored mitochondrial transcription factor A (TFAM) expression and attenuated activation of the PINK1/Parkin pathway, suggesting improved mitochondrial quality control [12]. At the molecular level, AM significantly reduced intracellular ROS accumulation, inhibited activation of c-Jun N-terminal kinase (JNK), restored PI3K/Akt signaling, suppressed Bax expression, preserved Bcl-2 levels, and reduced cleavage of caspase-3 and poly (ADP-ribose) polymerase (PARP). These findings indicate that AM exerts cytoprotective effects by simultaneously modulating oxidative stress, mitochondrial dysfunction, and apoptosis rather than targeting a single signaling pathway [43].

### 5.3. Glycerol-Induced Rhabdomyolysis AKI

Rhabdomyolysis-associated AKI develops secondary to myoglobin release, renal vasoconstriction, oxidative injury, and inflammatory activation. Compared with cisplatin nephrotoxicity, this model better reflects ischemic and pigment-induced renal injury frequently encountered in trauma and critical illness [3,13]. Eltahir et al. evaluated AM in a rat model of glycerol-induced rhabdomyolysis and demonstrated significant improvement in renal function following three days of treatment. AM reduced serum creatinine, BUN, renal lipid peroxidation, serum magnesium, TNF-α, and IL-6 while simultaneously improving renal histopathological injury. Importantly, the anti-inflammatory effects paralleled reductions in oxidative stress, further supporting the close interaction between these pathogenic pathways [13]. Although serum calcium concentrations remained unchanged, the overall improvement in renal histology suggests that modulation of inflammatory signaling rather than electrolyte homeostasis accounted for the beneficial effects observed in this model.

### 5.4. Current Evidence Gaps

Despite encouraging findings, the available literature remains limited in several important aspects. First, most studies have evaluated only short-term functional outcomes such as serum creatinine, BUN, oxidative stress biomarkers, and histological injury, whereas long-term renal recovery and progression to chronic kidney disease remain largely unexplored [4,11,12,13,14,21].

Second, mechanistic investigations have predominantly focused on oxidative stress, inflammation, and apoptosis. Mechanistic evidence regarding ferroptosis, pyroptosis, PANoptosis, immunometabolism, and cellular senescence remains limited or absent in kidney-specific AM studies [31]. Although these pathways are increasingly recognized as important contributors to AKI pathogenesis, their involvement in AM-mediated renoprotective effects remains hypothetical. Mitochondrial quality control has received some experimental support in cisplatin-induced nephrotoxicity; however, these findings require independent validation across additional AKI models.

Third, substantial heterogeneity exists regarding AM dosage, treatment duration, animal species, administration routes, and experimental protocols, limiting direct comparison among studies and complicating dose translation to human clinical trials [14]. Finally, no completed randomized clinical trial has yet evaluated AM in patients with AKI, indicating that current evidence remains confined to preclinical investigation [14,15,21].

Collectively, these observations suggest that although the preclinical evidence supporting AM is promising, additional mechanistic studies and carefully designed translational investigations are required before clinical application can be considered.

## 6. Translational Challenges and Future Perspective

### 6.1. Why Has AM Not Reached Clinical Practice?

Despite preliminary evidence of renoprotective effects in a limited number of experimental studies, AM has not yet been evaluated in clinical trials for AKI. This discrepancy between encouraging preclinical findings and the absence of clinical application reflects a common challenge in translational nephrology. Many interventions that demonstrate efficacy in experimental AKI ultimately fail to improve patient outcomes because of biological complexity, pharmacokinetic limitations, and differences between animal models and human disease [15]. Experimental AKI models are generally highly standardized and involve young, healthy animals exposed to a single renal insult, such as cisplatin administration or ischemia–reperfusion injury. In contrast, human AKI usually develops in elderly patients with multiple comorbidities, including chronic kidney disease, diabetes mellitus, cardiovascular disease, and sepsis. Furthermore, several pathogenic mechanisms often coexist simultaneously, making clinical AKI substantially more heterogeneous than experimental models [3,15,16]. These differences should be carefully considered when interpreting the promising renoprotective effects observed with AM in experimental studies.

### 6.2. Pharmacokinetic Limitations

One of the principal obstacles to clinical translation is the unfavorable pharmacokinetic profile of AM. The compound exhibits poor aqueous solubility, limited oral bioavailability, rapid glucuronidation, and variable systemic exposure following oral administration [34,37]. Low plasma concentrations may prevent achievement of therapeutic levels within injured renal tissue despite favorable biological activity observed in vitro. Beyond systemic bioavailability, kidney-specific drug disposition represents an important but largely unexplored translational challenge. Intestinal transport studies have identified both free AM and phase II metabolites following transepithelial transport [44]. However, effective renoprotection requires adequate exposure within the renal compartments involved in AKI, particularly tubular epithelial cells and the tubulointerstitial space. Such exposure may be influenced by glomerular filtration, plasma protein binding, tubular secretion and reabsorption, transporter-mediated uptake and efflux, and renal tissue partitioning.

Currently, direct data regarding the renal clearance, urinary excretion, tubular transporter-mediated handling, intracellular tubular accumulation, and renal interstitial distribution of AM and its metabolites are lacking. Therefore, plasma concentrations alone may not accurately reflect pharmacologically active AM exposure within injured renal compartments. Future pharmacokinetic studies should quantify AM and its major metabolites in plasma, urine, and renal tissue and, where technically feasible, characterize their distribution within tubular and interstitial compartments. These studies are required to determine whether pharmacologically relevant renal exposure can be achieved without excessive tissue accumulation or local toxicity. Consequently, optimization of AM delivery remains an important area of investigation. Liposomal formulations, polymeric nanoparticles, nanoemulsions, solid lipid nanoparticles, and phospholipid complexes have been investigated as strategies to improve aqueous solubility, systemic exposure, circulation time, and tissue delivery [34]. However, their ability to achieve targeted and therapeutically relevant AM exposure within injured renal compartments remains to be established.

### 6.3. Precision Medicine and Biomarker-Guided Therapy

The clinical and biological heterogeneity of AKI may contribute to variable treatment responses. Biomarker-guided approaches could potentially improve patient selection and help define the timing of AM administration. However, AKI biomarkers reflect different biological processes and phases of injury and should not be interpreted interchangeably. Stress biomarkers, such as urinary [TIMP-2]·[IGFBP7], may identify patients at risk of moderate-to-severe AKI before overt functional decline and could define a potential window for preventive or very early intervention [44]. Tubular injury biomarkers, including NGAL and KIM-1, may increase before serum creatinine and could identify subclinical or early AKI [44,45]. In contrast, serum creatinine, urine output, and cystatin C primarily reflect changes in kidney function and may be more useful for identifying established AKI and monitoring kidney recovery [1,2,44,45,46]. Serial assessment of injury and functional biomarkers may also help distinguish resolving injury from persistent kidney dysfunction. A potential biomarker-guided framework for AM could include risk enrichment using stress biomarkers, early intervention guided by tubular injury biomarkers, and monitoring of treatment response and kidney recovery using serial injury and functional markers. However, biomarker kinetics vary according to AKI etiology, baseline kidney function, clinical setting, specimen type, and assay platform; therefore, universal detection windows cannot be defined. Moreover, no study has evaluated biomarker-guided AM administration. This framework remains hypothesis-generating and requires prospective validation.

### 6.4. Comparison with Current AKI Management and Potential Combination Strategies

Current AKI management remains primarily supportive, as no pharmacological therapy has consistently demonstrated disease-modifying efficacy in established AKI. Standard care includes treatment of the underlying cause, optimization of hemodynamics and volume status, avoidance or dose adjustment of nephrotoxic medications, correction of metabolic complications, and kidney replacement therapy when indicated [1,2,3].

Sodium–glucose cotransporter-2 (SGLT2) inhibitors have demonstrated substantial kidney-protective effects and are associated with a reduced risk of AKI in patients with diabetes, chronic kidney disease, and heart failure [47]. Their proposed protective mechanisms include restoration of tubuloglomerular feedback, reduction in intraglomerular pressure, improved renal oxygen utilization, and attenuation of inflammation and oxidative stress [46]. However, these benefits primarily relate to AKI risk reduction and long-term kidney protection rather than treatment of established AKI. Therefore, SGLT2 inhibitors are not currently considered disease-modifying therapies for active AKI.

Experimental studies suggest that AM may modulate oxidative stress, inflammation, mitochondrial dysfunction, and apoptotic signaling. However, these findings remain preclinical, and no direct comparisons between AM and current AKI management or other kidney-protective therapies, including SGLT2 inhibitors, are available. AM should therefore not be considered equivalent to, superior to, or a replacement for, standard supportive care. Given its proposed multi-pathway effects, AM may be investigated as an adjunct to supportive or etiology-specific treatment. Combination with kidney-protective agents, including SGLT2 inhibitors, may be of future interest because of potentially complementary effects on renal hemodynamics, metabolism, oxidative stress, and inflammation. However, this concept remains hypothetical, and no experimental or clinical studies have evaluated AM–SGLT2 inhibitor combination therapy in AKI.

### 6.5. Artificial Intelligence-Assisted Drug Discovery and Systems Pharmacology

Recent advances in systems biology provide new opportunities to accelerate development of AM as a therapeutic agent. Network pharmacology studies demonstrate that AM interacts with numerous molecular targets involved in oxidative stress, inflammatory signaling, apoptosis, and mitochondrial homeostasis [42]. Artificial intelligence, machine learning, molecular docking, and molecular dynamics simulations may further identify previously unrecognized therapeutic targets, optimize compound modification, predict toxicity, and facilitate drug repurposing. Integration of computational biology with experimental validation may substantially accelerate translational development.

### 6.6. Potential Applicability to Other AKI Phenotypes

AKI is a heterogeneous clinical syndrome, and the mechanisms and relative contributions of tubular, vascular, inflammatory, and metabolic injury vary according to etiology. Therefore, the renoprotective effects of AM observed predominantly in cisplatin-induced nephrotoxicity cannot be assumed to apply uniformly to other forms of AKI.

Sepsis-associated AKI is characterized by dysregulated inflammation, endothelial and microcirculatory dysfunction, mitochondrial injury, oxidative stress, and metabolic reprogramming rather than renal hypoperfusion alone. These mechanisms partially overlap with pathways modulated by AM, including NF-κB-mediated inflammation, NLRP3 inflammasome activation, oxidative stress, and mitochondrial dysfunction. In support of this mechanistic plausibility, AM has been shown to promote autophagy, suppress NLRP3 inflammasome activation in lipopolysaccharide-stimulated macrophages, and improve survival in a murine cecal ligation and puncture model of sepsis [48,49]. However, that study did not establish kidney-specific efficacy, and no dedicated study has evaluated AM in sepsis-associated AKI. Thus, its potential benefit in this phenotype remains hypothetical. Renal ischemia–reperfusion injury similarly involves excessive mitochondrial ROS generation, ATP depletion, endothelial dysfunction, innate immune activation, apoptosis, ferroptosis, and necroptosis [50]. The antioxidant, mitochondrial-preserving, anti-inflammatory, and anti-apoptotic effects reported for AM in cisplatin nephrotoxicity could therefore be relevant to ischemic AKI. AM has also attenuated oxidative stress and tissue injury in experimental cardiac reperfusion injury, providing indirect evidence that it may modulate conserved reperfusion-associated pathways. Nevertheless, direct evidence in renal ischemia–reperfusion models is currently lacking.

Overall, the overlap in downstream mechanisms provides a rationale for investigating AM in sepsis-associated and ischemia–reperfusion AKI, but it should not be interpreted as evidence of efficacy across these phenotypes. Future studies should use clinically relevant models and assess renal function, tubular and endothelial injury, microcirculatory alterations, mitochondrial bioenergetics, regulated cell death, and long-term kidney recovery.

### 6.7. SWOT Analysis of AM as a Potential Therapeutic Strategy for AKI

To provide a structured assessment of the translational potential of AM in AKI, its current strengths, weaknesses, opportunities, and threats are summarized in Table 2. The principal strengths of AM include its ability to modulate several interconnected pathways implicated in AKI, particularly oxidative stress, inflammatory signaling, mitochondrial dysfunction, and apoptosis, together with the consistent renoprotective effects observed in the available experimental studies. However, these findings should be interpreted in the context of important weaknesses, including the small and predominantly preclinical evidence base, reliance on cisplatin-induced nephrotoxicity models, poor oral bioavailability, uncertain kidney-specific pharmacokinetics, and an incompletely characterized safety profile. Several opportunities may support further development, including advanced drug-delivery systems, validation in clinically relevant AKI models, kidney-targeted formulations, biomarker-guided patient selection, and carefully designed early-phase clinical studies. Conversely, major threats include the biological heterogeneity of human AKI, uncertain reproducibility across different AKI phenotypes, potential dose-dependent toxicity and off-target effects, regulatory challenges related to formulation standardization, and the possibility that experimental efficacy may not translate into clinical benefit. This analysis highlights the need for a staged translational approach that prioritizes mechanistic validation, pharmacokinetic and toxicological characterization, and reproducibility across diverse AKI models before clinical efficacy studies are considered.

### 6.8. Future Research Priorities

Several important knowledge gaps should be addressed before AM can be translated into clinical nephrology. First, future studies should investigate emerging mechanisms including ferroptosis, pyroptosis, PANoptosis, immunometabolism, mitochondrial quality control, and cellular senescence. Second, standardized pharmacokinetic and dose-escalation studies are required to determine clinically relevant exposure and safety. Third, multicenter preclinical studies using aged animals and clinically relevant AKI models are needed to improve external validity. Finally, early-phase clinical trials should evaluate safety, tolerability, pharmacokinetics, biomarker responses, and preliminary efficacy in patients at high risk of AKI, particularly those undergoing cardiac surgery, receiving nephrotoxic chemotherapy, or developing sepsis-associated AKI. Collectively, these investigations will determine whether AM can progress from an experimentally promising phytochemical toward a clinically applicable disease-modifying therapy for AKI.

## 7. Conclusions

AKI remains a major global health challenge with no established disease-modifying pharmacological therapy. Current experimental evidence suggests that AM may attenuate kidney injury through modulation of oxidative stress and inflammatory signaling, preservation of mitochondrial function, and attenuation of apoptosis-associated pathways. However, these findings are derived from a small and heterogeneous body of preclinical evidence, predominantly involving cisplatin-induced nephrotoxicity. Evidence from other clinically relevant AKI phenotypes, including sepsis-associated and ischemia–reperfusion AKI, remains limited or absent. Furthermore, direct evidence linking AM to ferroptosis, pyroptosis, PANoptosis, immunometabolism, cellular senescence, and kidney repair is limited, and these pathways should be considered mechanistic hypotheses rather than established mechanisms of AM-mediated renoprotective effects.

Several major limitations currently restrict the clinical translation of AM. Experimental studies vary in model selection, dose, route of administration, treatment timing, and outcome assessment, limiting direct comparison and dose translation. Most studies have evaluated short-term functional, biochemical, and histological outcomes, whereas long-term kidney recovery and the AKI-to-CKD transition remain insufficiently studied. Poor aqueous solubility limited oral bioavailability, extensive metabolism, uncertain kidney-specific pharmacokinetics and intrarenal exposure, and an incompletely characterized therapeutic window and safety profile represent additional barriers. Importantly, no clinical study has evaluated the efficacy or safety of purified AM in patients with AKI.

Overall, AM should currently be regarded as an investigational compound with preliminary preclinical renoprotective potential rather than an established or clinically translatable therapy. Its future development should follow a staged research pathway. First, the proposed mechanisms and renoprotective effects should be independently validated in diverse and clinically relevant AKI models, including sepsis-associated and ischemia–reperfusion AKI, as well as aged and comorbidity-enriched models incorporating diabetes, pre-existing CKD, or other clinically relevant chronic conditions. Second, standardized dose–response, repeated-dose toxicity, and pharmacokinetic studies in models with normal and impaired kidney function are required to characterize intrarenal exposure, potential tissue accumulation, drug–drug interactions, and an acceptable safety margin. Third, optimized formulations, treatment timing, long-term kidney recovery, and reproducibility should be evaluated in translational models. Finally, carefully designed early-phase clinical studies should be considered only after adequate preclinical efficacy, pharmacokinetic, and safety evidence has been established. This staged approach may provide a more rigorous framework for determining whether further clinical development of AM in AKI is justified.

## Figures and Tables

**Table 1:** Summary of experimental evidence evaluating AM in acute kidney injury.

Study	Model	AM Dose	Route and Treatment Schedule	Biomarkers/Outcomes Assessed	Main Findings and Proposed Mechanisms	Key Limitations
Pérez-Rojas et al., 2009 [11]	Cisplatin-induced nephrotoxicity; rat model	12.5 mg/kg	Intraperitoneal administration; AM administered before and during cisplatin exposure	Serum creatinine, BUN, creatinine clearance, renal histopathology, lipid peroxidation, protein carbonylation, nitrosative stress, catalase activity, inflammatory and fibrotic markers	Improved renal function and attenuated tubular injury; reduced oxidative/nitrosative stress; preserved catalase activity; reduced inflammatory and fibrotic responses	Single nephrotoxic model; short-term outcomes; preventive treatment design; no long-term renal outcomes or clinical validation
Reyes-Fermín et al., 2019 [12]	Cisplatin-induced tubular injury; LLC-PK1 proximal tubular cells	4 μM	AM pretreatment before cisplatin exposure; in vitro experimental protocol	Cell viability, mitochondrial respiration, mitochondrial membrane potential, ATP-related mitochondrial function, ROS, mitochondrial dynamics, TFAM, PINK1/Parkin-related signaling, and cellular injury markers	Preserved mitochondrial function and bioenergetics; reduced oxidative injury and excessive mitochondrial stress responses	In vitro model only; single cell line; preventive treatment design; uncertain in vivo exposure and dose translation
Li et al., 2020 [43]	Cisplatin-induced tubular cell injury; HEK293 cells	5–40 μM	AM pretreatment followed by cisplatin exposure for 24 h	Cell viability, MDA, glutathione, intracellular ROS, apoptosis, PI3K/Akt signaling, JNK signaling, Bax, Bcl-2, caspase-3, and PARP	Increased cell viability; reduced oxidative stress and apoptosis; modulation of PI3K/Akt and JNK signaling	In vitro model only; HEK293 cells may not fully represent differentiated renal tubular cells; preventive treatment design; uncertain clinically achievable concentrations and renal tissue exposure
Eltahir et al., 2023 [13]	Glycerol-induced rhabdomyolysis-associated AKI; rat model	175 mg/kg/day	Intraperitoneal administration once daily for 3 days after glycerol-induced injury	Serum creatinine, BUN, renal histopathology, lipid peroxidation, TNF-α, IL-6, serum calcium, and magnesium	Improved renal function and histopathological injury; reduced lipid peroxidation and circulating TNF-α and IL-6	Single animal model; short treatment and observation period; high experimental dose; limited mechanistic biomarkers; no long-term kidney recovery or dose–response assessment
Chatatikun et al., 2025 [14]	Multiple experimental AKI models; systematic review and meta-analysis	Varied across included studies	Varied routes, timing, and treatment durations across included studies	Serum creatinine, BUN, oxidative stress biomarkers, inflammatory mediators, and renal histopathology	Pooled evidence suggested improvements in renal function, oxidative stress, inflammation, and histological injury	Small preclinical evidence base; heterogeneity in models, doses, routes, and treatment protocols; predominantly animal and in vitro evidence; possible publication bias; no human clinical data
Chatatikun et al., 2026 [42]	AKI target prediction; network pharmacology and molecular docking	Not applicable	Not applicable	Predicted AM–AKI targets, protein–protein interaction networks, pathway enrichment, and molecular docking	Identified potential targets related to oxidative stress, inflammation, apoptosis, PI3K/Akt, MAPK, and NF-κB signaling	Computational predictions only; no direct assessment of renal function or kidney injury biomarkers; requires experimental and clinical validation

**Table 2:** SWOT analysis of AM as a potential therapeutic strategy for AKI.

Strengths	Weaknesses	Opportunities	Threats
Multi-pathway activity involving oxidative stress, inflammation, mitochondrial dysfunction, and apoptosis	Limited number of experimental studies	Validation in sepsis-associated and ischemia–reperfusion AKI models	Biological and clinical heterogeneity of human AKI
Renoprotective effects observed across available experimental studies	Evidence derived predominantly from cisplatin-induced nephrotoxicity models	Development of kidney-targeted drug-delivery systems	Limited reproducibility across different AKI phenotypes
Modulation of interconnected pathways involved in AKI pathogenesis	Lack of human efficacy and safety data	Development of liposomal, nanoparticle, and other advanced formulations	Potential dose-dependent toxicity and off-target effects
Preliminary evidence supporting mitochondrial preservation	Poor aqueous solubility and limited oral bioavailability	Pharmacokinetic characterization and dose optimization	Potential drug–drug interactions in patients with AKI and polypharmacy
Potential compatibility with advanced drug-delivery approaches	Extensive metabolism and variable systemic exposure	Biomarker-guided patient selection and treatment	Failure of experimental efficacy to translate into clinical benefit
Established pharmacological interest as a naturally derived bioactive compound	Uncertain kidney-specific pharmacokinetics and intrarenal distribution	Evaluation of long-term kidney recovery and the AKI-to-CKD transition	Difficulty achieving therapeutically relevant renal exposure

## Data Availability

No new data were created or analyzed in this study. Data sharing is not applicable to this article.

## Abbreviations

The following abbreviations are used in this manuscript:

AKI	Acute kidney injury
CKD	Chronic kidney disease
AM	Alpha-mangostin
ROS	Reactive oxygen species
Nrf2	Nuclear factor erythroid 2-related factor 2
Keap1	Kelch-like ECH-associated protein 1
NQO1	NAD(P)H quinone oxidoreductase 1
GPx	Glutathione peroxidase
SOD	Superoxide dismutase
NF-κB	Nuclear factor-kappa B
MAPK	Mitogen-activated protein kinase
NLRP3	NOD-like receptor family pyrin domain-containing 3
TNF-α	Tumor necrosis factor-alpha
IL	Interleukin
MCP-1	Monocyte chemoattractant protein-1
TLR	Toll-like receptor
DAMP	Damage-associated molecular pattern
ATP	Adenosine triphosphate
PINK1	PTEN-induced kinase 1
TFAM	Mitochondrial transcription factor A
RIPK	Receptor-interacting protein kinase
MLKL	Mixed lineage kinase domain-like protein
GPX4	Glutathione peroxidase 4
PARP	Poly(ADP-ribose) polymerase
JNK	c-Jun N-terminal kinase
PI3K	Phosphoinositide 3-kinase
Akt	Protein kinase B
AMPK	AMP-activated protein kinase
COX-2	Cyclooxygenase-2
iNOS	Inducible nitric oxide synthase
BUN	Blood urea nitrogen
NGAL	Neutrophil gelatinase-associated lipocalin
KIM-1	Kidney injury molecule-1
DKK3	Dickkopf-3
SGLT2	Sodium-glucose cotransporter-2
